# Perioperative oxygen therapy: a protocol for an overview of systematic reviews and meta-analyses

**DOI:** 10.1186/s13643-022-02005-3

**Published:** 2022-07-12

**Authors:** Adel Elfeky, Yen-Fu Chen, Amy Grove, Amy Hooper, Anna Wilson, Keith Couper, Marion Thompson, Olalekan Uthman, Rachel Court, Sara Tomassini, Joyce Yeung

**Affiliations:** 1 Warwick Medical School, University of Warwick, Coventry, CV4 7AL UK; 2 University Hospitals Birmingham NHS Foundation Trust, Birmingham, UK; 3 Independent patient and public involvement and engagement advisor, Birmingham, UK

## Abstract

**Background:**

Oxygen is routinely given to patients during and after surgery. Perioperative oxygen administration has been proposed as a potential strategy to prevent and treat hypoxaemia and reduce complications, such as surgical site infections, pulmonary complications and mortality. However, uncertainty exists as to which strategies in terms of amount, delivery devices and timing are clinically effective. The aim of this overview of systematic reviews and meta-analyses is to answer the research question, ‘For which types of surgery, at which stages of care, in which sub-groups of patients and delivered under what conditions are different types of perioperative oxygen therapy clinically effective?’.

**Methods:**

We will search key electronic databases (MEDLINE, EMBASE, the Cochrane Database of Systematic Reviews, CENTRAL, Epistemonikos, PROSPERO, the INAHTA International HTA Database and DARE archives) for systematic reviews and randomised controlled trials comparing perioperative oxygen strategies.

Each review will be mapped according to type of surgery, surgical pathway timepoints and clinical comparison. The highest quality reviews with the most comprehensive and up-to-date coverage of relevant literature will be chosen as anchoring reviews. Standardised data will be extracted from each chosen review, including definition of oxygen therapy, summaries of interventions and comparators, patient population, surgical characteristics and assessment of overall certainty of evidence. For clinical outcomes and adverse events, the overall pooled findings and results of subgroup and sensitivity analyses (where available) will be extracted. Trial-level data will be extracted for surgical site infections, mortality, and potential trial-level effect modifiers such as risk of bias, outcome definition and type of surgery to facilitate quantitative data analysis. This analysis will adopt a multiple indication review approach with panoramic meta-analysis using review-level data and meta-regression using trial-level data. An evidence map will be produced to summarise our findings and highlight any research gaps.

**Discussion:**

There is a need to provide a panoramic overview of systematic reviews and meta-analyses describing peri-operative oxygen practice to both inform clinical practice and identify areas of ongoing uncertainty, where further research may be required.

**Systematic review registration:**

PROSPERO CRD42021272361

**Supplementary Information:**

The online version contains supplementary material available at 10.1186/s13643-022-02005-3.

## Background

Patients who require surgery routinely receive supplemental oxygen both during (intraoperative) and following (postoperative) surgery. They may also receive supplemental oxygen prior to surgery (preoperative). The aim of oxygen administration in the perioperative setting is the prevention or treatment of hypoxaemia and to reduce the risk of both operative and post-operative complications. Perioperative hypoxaemia is common [1–4] and increases the risk of cardiopulmonary complications [5, 6], delirium [7], prolonged hospital stay [1, 4] and mortality [8]. However, liberal use of supplemental oxygen leading to hyperoxaemia may have harmful effects mediated by increased reactive oxygen species generation, hyperoxic vasoconstriction and decreased ventilation. These effects are associated with reduced cardiac, pulmonary and renal blood flow, atelectasis, respiratory complications and higher mortality [9–12].

The World Health Organisation (WHO) guidelines recommend that adults undergoing general anaesthesia with tracheal intubation for surgical procedures should receive high concentration oxygen (80%) intraoperatively and postoperatively for 2–6 h [13]. However, this recommendation has generated substantial controversy and there is ongoing debate surrounding the overall safety of using high concentrations of oxygen [9, 14–16]. The recommendation is not supported by clinical guidelines issued by other organisations [17]. This uncertainty has led to a lack of standardised approach in perioperative oxygen therapy and marked variability in the care of patients undergoing surgery [14].

A further consideration particularly post-operatively and during surgery where patients are not intubated is the use of different oxygen delivery devices. Non-invasive respiratory support strategies, such as continuous positive airway pressure (CPAP), non-invasive positive pressure ventilation (NIPPV), and high-flow nasal oxygen (HFNO), have been proposed to improve oxygenation in hypoxaemic patients and to reduce the risk of post-surgical complications [18]. There are potential physiological benefits of these strategies through improved lung compliance and alveolar recruitment [19], but may also cause harm through both volutrauma and barotrauma [20, 21]. Randomised controlled trials (RCTs) evaluating the use of these strategies have produced conflicting results [22].

The clinical effectiveness of perioperative oxygen therapy in improving patient outcomes is a highly complex area of investigation. The effect may differ in patient groups such as age groups (adult versus children versus neonates), different underlying condition (cancer versus non-cancer), different types of anaesthesia (general anaesthesia, regional anaesthesia, or sedation) or different types of surgery (e.g. cardiothoracic surgery, trauma, elective, or emergency abdominal surgery). A large number of systematic reviews have explored the use of different oxygen strategies in the peri-operative setting [23–36]. Our initial scoping of systematic reviews suggests substantial overlapping in their evidence coverage. These reviews covered different phases and timepoints in the surgical pathway, oxygenation strategies, oxygen delivery devices, and clinical conditions. Differences in setting, strategy, population, and outcome may explain some of the variability in findings of these reviews. On this basis, an overview of systematic reviews and panoramic meta-analyses is needed to map, synthesise and assess the reliability of evidence from systematic reviews on the clinical effectiveness of different types of perioperative oxygen therapy strategies across all patient groups and surgical settings. Bringing together the available evidence will aid clinical decision-making and importantly highlight the specific areas in which further high-quality research is required.

### Aim and objectives

#### Aim

To conduct an overview of systematic reviews and meta-analyses to answer the research question:

For which types of surgery, at which stages of care, in which sub-groups of patients and delivered under what conditions are different types of perioperative oxygen therapy clinically effective?

#### Objectives

1. To assess the volume and quality of evidence on perioperative oxygen therapy across different clinical areas through systematically identifying, mapping and summarising available systematic reviews of RCTs.
2. To conduct panoramic meta-analyses of the clinical effectiveness of perioperative oxygen therapy across clinical areas by common outcomes.
3. To formulate research recommendations by identifying areas of clinical uncertainty where there is either no evidence or insufficient evidence to inform clinical decision-making.

#### Advisory panel and patient and public involvement

An advisory panel will represent key stakeholders involved in the use of perioperative oxygen, including patients and a multidisciplinary group of clinical specialists with expertise in anaesthesia, critical care, surgery, and physiotherapy. Panel members will review mapping of studies, synthesis strategy, and interpretation of evidence to guide the production of clinically relevant recommendations and conclusions. In addition, a patient representative (MT) will work closely with the research team throughout the project.

## Methods

This protocol is reported in consultation with the PRISMA-P statement [37]. The PRISMA-P checklist is provided as Additional file 1. Any amendments to the protocol until completion of the overview shall be provided with reasons and will be available to public view. We will follow the Preferred Reporting Items for Overviews of Reviews (PRIOR) guidelines for overviews of reviews of healthcare interventions [38] if they become available during our project timeframe or use the updated PRISMA guideline [39] for reporting the review.

### Study selection criteria

The inclusion criteria are as follows:

(i) *Patients*: hospitalised patients undergoing surgical procedures (where patients would normally be provided with anaesthesia by either an anaesthetist or a qualified anaesthetic practitioner) of any age group, and surgical specialty at any stage of the surgical pathway including preoperative, intraoperative, and postoperative periods.
(ii) *Intervention*: perioperative oxygen therapy, defined as oxygenation strategy where the primary purpose of the intervention is to optimise oxygenation/oxygen delivery, with the aim of preventing hypoxaemia or reducing complications during the perioperative period. Our review will exclude systematic reviews that primarily focus on intraoperative ventilation strategies (e.g. ventilatory rate, pressure and volume settings), hyperbaric oxygen therapy and extracorporeal life support [40]. Reviews that examine pre-oxygenation strategies for tracheal intubation will also be excluded. To include all relevant reviews, we will not use predefined arbitrary thresholds or targets of oxygenation.
(iii) *Comparator*: any comparator or control.
(iv) *Outcomes*: Primary outcomes (for selecting reviews to be included in panoramic meta-analysis and RCTs to be included in meta-regression)

1. Surgical site infection within 30 days of follow-up after surgery—we will follow definitions of the US Centers for Disease Control and Prevention (CDC) where possible. Reviews (and RCTs included in the reviews) that have adopted other definitions will still be included and examined if meeting other inclusion criteria, but differences in the outcome definitions will be recorded and highlighted.
2. All-cause mortality within 30-days postoperatively.

Secondary outcomes (for other reviews to be narratively synthesised)

1. Postoperative pulmonary complications (PPC): defined according to the most recent consensus definition of PPC [41] as composite of respiratory diagnoses: (i) atelectasis detected on computed tomography or chest radiograph, (ii) pneumonia using US CDC criteria, (iii) Acute Respiratory Distress Syndrome (ARDS) using Berlin consensus definition, (iv) pulmonary aspiration (clear clinical history AND radiological evidence).
2. Postoperative respiratory failure: Including ARDS defined using Berlin consensus definition [42] and need for mechanical ventilation.
3. Definitions for the above outcomes are recommended by the StEP-COMPAC Group [41]. We will accept similar outcomes defined differently in previous studies. Differences in the outcome definitions will be recorded and highlighted.
4. Mortality up to the longest point of post-operative follow-up
5. Length of hospital stay: the number of days from the day of surgery to hospital discharge or death.
6. Intensive care unit (ICU) admission: unplanned admission to ICU within 14 days of surgery.
7. Quality of life.

Systematic reviews that cover any of the primary or secondary outcomes listed above are potentially eligible. Further outcomes not listed above but are identified during the course of the review and considered important by the Advisory Group may be examined. The post hoc addition of such outcomes will be explicitly stated.

(v) *Study design*: systematic reviews and meta-analyses of RCTs that examine the use of perioperative oxygen therapy. We will include systematic reviews that include both randomised and non-randomised studies as long as evidence summarised from RCTs is reported separately. To be included, systematic reviews must fulfil a minimum of four methodological criteria as defined by Centre for Reviews and Dissemination, University of York guidance [43], specifically they must report inclusion/exclusion criteria of studies, an adequate search strategy, synthesis of included studies, description of and quality assessment of included studies.

### Information sources and search strategy

Our search will be developed and conducted by an information specialist according to the principles of the Cochrane Handbook for Systematic Reviews of Interventions and recommendations for conducting Overviews of Systematic Reviews [44]. Relevant reviews will be identified using index terms and text words related to oxygen therapies and surgeries through searches of key electronic databases including MEDLINE EMBASE, the Cochrane Database of Systematic Reviews, EPistemonikos [45], PROSPERO [46], the INAHTA International HTA Database, and the DARE archives. The final strategy is included in Additional file 2. Searches will not be limited by date or publishing language. Non-English language articles will be translated into English. References will also be located through a review of reference lists for relevant articles and through use of citation search facilities provided by the Web of Knowledge. In addition, systematic searches of systematic review registries and the Internet using the Google.co.uk search engine will be conducted to identify unpublished materials and work in progress. In order to ensure that emerging evidence is covered, we will also search for recently published or ongoing/planned RCTs in the Cochrane CENTRAL database and major clinical trial registries for the recent years (the exact time periods will depend on the timing when the searches were performed in published systematic reviews) and will set up citation alerts in MEDLINE and EMBASE (based on the CENTRAL search strategy, but with the addition of search filters for RCTs).

### Study selection and mapping

#### Initial review selection

**Fig. 1:**
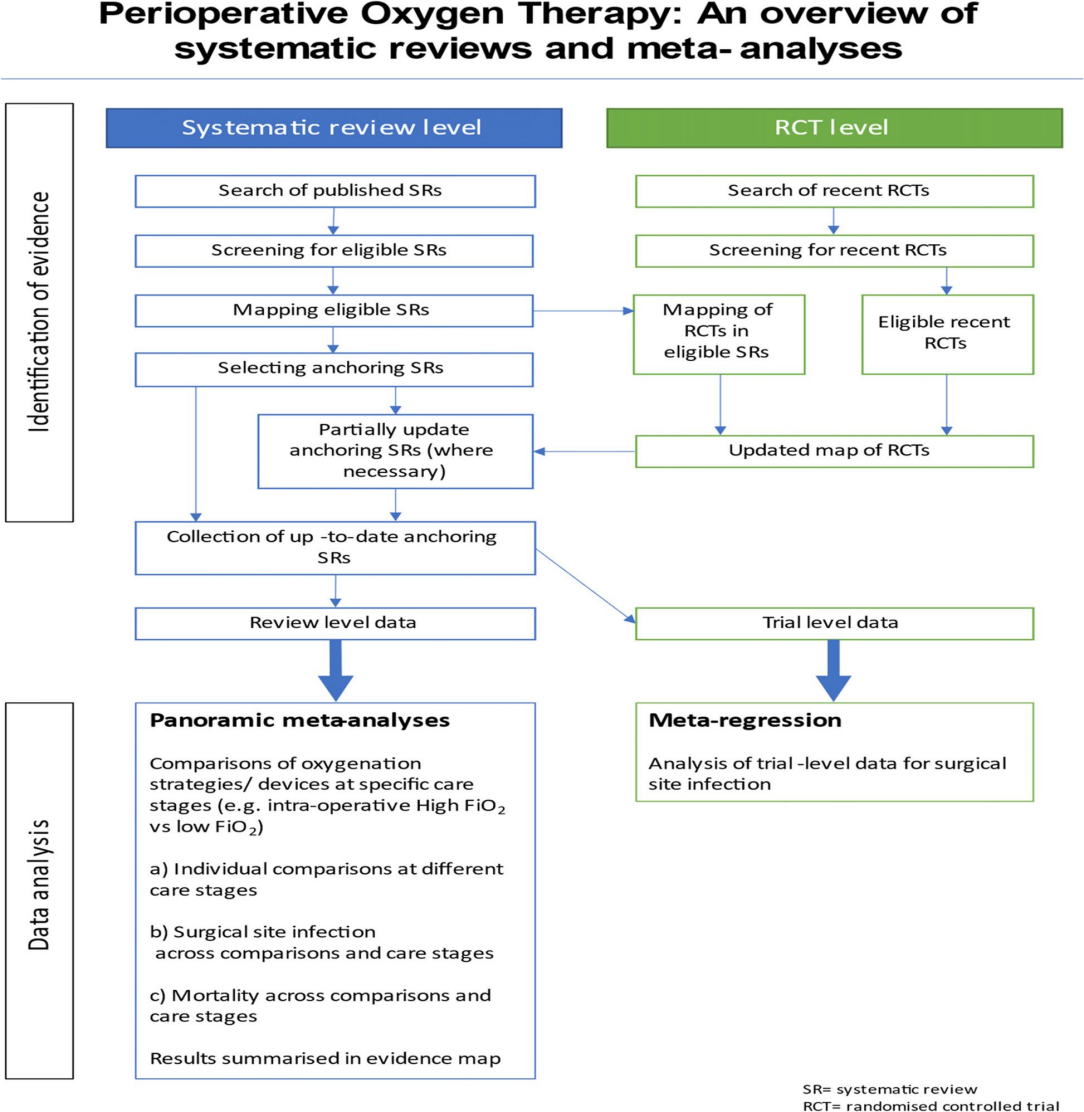
Overview schema

Titles and abstracts of records retrieved will be screened by two reviewers independently, disagreement will be resolved by discussion or if needed with the input of a third senior reviewer. Full-text articles considered potentially meeting inclusion criteria will be assessed for inclusion by two reviewers independently and disagreements resolved as above. Figure 1 illustrates the overview schema. Records retrieved from the Cochrane CENTRAL database and clinical trial registries will go through the same screening and selection process as above. We will use Evidence for Policy and Practice Information (EPPI)-Reviewer 4 software to manage records and data throughout the review.

#### Study mapping

Following confirmation of eligibility, each review will be mapped according to types of surgery, stages of perioperative care and comparisons made (e.g. between different oxygenation strategies or delivery device). We will map all RCTs included in the systematic reviews to gauge the extent of overlap between reviews and to ensure that no double counting of evidence will occur when undertaking further quantitative analyses. Where reviews covering largely overlapping topic areas and RCTs are identified, a single review with the most comprehensive coverage of literature and/or the highest methodological quality, as judged by ROBIS tool [47], will be selected (with advice from the advisory panel) as the anchoring review. Where reviews overlap only partially in their scope or evidence coverage, multiple reviews may be retained as anchoring reviews. RCTs published since the anchoring systematic review searches that are judged to be eligible will be added to the RCT map.

Then chosen anchoring reviews will be checked against the completed RCT map to see if any important RCTs not included in the anchoring reviews warrant to be added by updating relevant analyses in the anchoring reviews (where this is considered necessary and feasible). We are aware that updating existing reviews is a significant undertaking and so the decision to (partially) update any anchoring reviews will be made judiciously in consultation with the advisory panel, taking into consideration the quality and sample sizes of the new RCTs and the certainty of evidence. Where new RCTs are identified or existing evidence base has changed (e.g. we are aware of previous RCTs having been retracted) [48, 49] but the new data/change have not been incorporated through partial update of the anchoring reviews, they will be highlighted alongside relevant anchoring reviews.

### Data extraction

Standardised data will be extracted by two independent reviewers, from each chosen anchoring systematic review. This will include:

1. Review characteristics: year published, country, number of RCTs included, number of patients, summary of intervention, and comparator.
2. Patient population: type of surgery, reported patient characteristics.
3. All clinical outcomes and adverse events reported.
4. Reported pooled results for the primary and secondary outcomes listed above, results of sub-group, and sensitivity analysis.
5. Certainty of evidence.
6. Risk of bias assessments and publication bias.

Some of the data will have been extracted during the stage of study mapping to inform the selection of anchoring reviews. Authors of the original reviews and RCTs will be contacted for missing data or data queries.

### Risk of bias assessment

Risk of bias for each of the systematic reviews will be assessed using the Risk of bias in Systematic Reviews (ROBIS) tool [47]. New RCTs used to update the anchoring reviews will be assessed using the appropriate Cochrane ROB2 tool for each relevant outcome. If systematic reviews have used different methodological approaches to assess the risk of bias that could impact on the comparability of findings between the reviews, the risk of bias for included RCTs will be reassessed for each relevant outcome using the appropriate ROB2 tool. All assessments will be conducted by two reviewers independently with conflicts resolved through discussion or consultation with a third reviewer. Risk of bias assessments will be presented in a tabular format.

### Data synthesis

The data synthesis will serve four purposes for this overview, each with a corresponding set of analyses:

1. Panoramic meta-analyses for individual comparisons to explore the effectiveness of perioperative oxygen therapy in different patient populations and settings;
2. Top level panoramic meta-analysis to test the scientific hypothesis that perioperative oxygen therapy reduces the risk of surgical site infection; [50]
3. Top level panoramic meta-analysis to evaluate the overall benefit/risk of perioperative oxygen therapy in terms of mortality across different clinical conditions and settings;
4. Meta-regression to explore potential trial level effect modifiers for surgical site infection among perioperative oxygen therapy trials.

Analyses will be performed in STATA [51] or WinBUGS as appropriate. Each of these sets of analysis are described in further details below.

### Panoramic meta-analysis for individual comparisons

These panoramic meta-analyses will be conducted for individual comparisons (e.g. high vs low FiO_2_ strategy during operation; HFNO vs conventional oxygen therapy post-operation) using pooled results from the meta-analysis already conducted in the anchoring reviews as the unit of analysis (or using subgroup data from the anchoring review or where necessary, a pooled subset of included trials if the anchoring review had wide coverage). For anchoring reviews that have been updated with new RCTs, a new meta-analysis will be conducted prior to panoramic meta-analysis. Risk ratios with 95% confidence intervals will be reported and presented as forest plots.

Each panoramic meta-analysis will be stratified by one subgroup feature (e.g. type of surgery). Potential subgroups may include:

- Type of surgery: surgical specialties such as cardiothoracic surgery, elective abdominal surgery (uncontaminated) versus emergency abdominal surgery (contaminated), trauma, joint replacement surgery etc.
- Type of underlying condition: cancer versus non-cancer
- Anaesthesia type: general anaesthesia versus regional anaesthesia versus sedation
- Patient age: adults versus children versus neonates
- Targeted use: preventive (preventing complications) versus therapeutic (treating hypoxaemia)
- Different certainty of evidence (based on GRADE assessment).

Judgements regarding what panoramic meta-analysis will be undertaken for what outcome using what subgrouping factors will be decided with advice from our advisory panel. We will take into account the availability of data and theoretical underpinning of the plausibility of the subgroup feature being a potential effect modifier for the specific outcome. We envisage that ‘type of surgery’ will be the subgroup feature used to stratify the panoramic meta-analysis in most cases.

For all panoramic meta-analyses, data judged to be of low risk of bias will be used in the main analysis and addition data of various levels of risk of bias will be included in sensitivity analyses. A random effects model will be used. Between-study and between-review heterogeneity will be estimated using the *I*^2^ statistic [52]. In the presence of a high level of statistical heterogeneity, the decision as to whether a pooled estimate will be calculated and presented will be guided by discussions with the review advisory panel.

The panoramic meta-analyses will be exploratory in nature and will be carefully interpreted as such. Where appropriate, we will examine the reliability and conclusiveness of the available evidence with the aid of trial sequential analyses [53, 54].

### Top level panoramic meta-analyses for surgical site infection and 30-day all-cause mortality

Two top level, exploratory panoramic meta-analyses are planned. These top level panoramic meta-analyses will further aggregate the data from individual panoramic meta-analysis across oxygenation strategies and surgical care stages, with pooled data from individual panoramic meta-analysis as the unit of analysis. The first top level panoramic meta-analysis will explore the effect of perioperative oxygen therapy on surgical site infection. Anticipating both clinical and statistical heterogeneity among the diverse evidence, the aim is not to generate an overall pooled effect estimate, which would be difficult to interpret. Instead, the main purpose is to enable a quantitative inspection of estimated treatment effects and level of heterogeneity across different stages of surgical care and oxygenation strategies, and to explore the compatibility of existing evidence against the hypothesis that perioperative exposure to higher levels of oxygen, as a whole, reduces surgical site infection.

A similar top level panoramic meta-analysis is planned to evaluate the overall benefit/risk of perioperative oxygen therapy on postoperative 30-day all-cause mortality. This analysis mirrors the analysis described above (further aggregation of data from individual panoramic meta-analysis).

We will proceed with these top level panoramic meta-analyses only if the levels of heterogeneity within and between individual panoramic meta-analyses are acceptable (i.e. not clearly showing opposite effects). Analyses will be performed using a random effects model. Heterogeneity between individual panoramic meta-analyses will be quantified using *I*^2^ statistic.

### Meta-regression

The panoramic meta-analyses are based on review-level pooled data. As such they may be subject to bias and confounding arising from different characteristics and methods between reviews. In order to minimise this and to explore potential effect modifiers further, an additional analysis of surgical site infection data using meta-regression approach will be undertaken, with individual trials included in the panoramic meta-analyses (which draw from updated anchoring reviews) as the unit of analysis. This will enable better exploration of effect modifiers and adjustment for potential confounders (e.g. risk of bias and prophylactic antibiotic) at study level. Given the need for data from individual trials which may not have been extracted/presented in the original systematic reviews, the coverage of RCTs in the meta-regression will be partly dependent on the total number of RCTs identified (feasibility due to volume of evidence) and reporting of relevant data in individual trials (availability of suitable data). Variables to be included will be chosen from important subgrouping variables explored in individual panoramic meta-analyses above and other potential effect modifiers highlighted in the literature. The list of variables will be determined a priori before the meta-regression is carried out to ensure that the analysis is theory-driven rather than data-driven. As the number of studies reporting mortality outcome and the number of death events will be substantially smaller than those of surgical site infections, no meta-regression analysis is planned for this outcome as available data are unlikely to provide sufficient statistical power.

### Publication bias/issue of selective reporting

Information concerning publication bias and selective outcome reporting at trial level will be extracted from selected anchoring reviews and presented within the summary of each review. At review level, selective outcome reporting will be assessed by comparing outcomes presented in the published reviews against outcomes specified in its protocol (where available). While publication bias related to RCTs has been well documented, we are not aware of evidence demonstrating selective publication of systematic reviews with positive or statistically significant findings or reliable methods for assessing this. However, we will highlight where there is no evidence of pre-registration for identified systematic reviews and explore relevant systematic reviews which have been registered but not subsequently published by contacting authors to clarifying reasons for non-publication.

### Assessment of the certainty in evidence

The GRADE approach will be used to assess the certainty of evidence for each outcome within each (updated) anchoring reviews [55]. This will be taken directly from the reviews if already reported in the anchoring reviews which do not require updating; otherwise, the GRADE assessment for the updated anchoring reviews will be undertaken by two researchers independently.

### Presentation of findings

We will produce an evidence map [56, 57] to present the volume of evidence across all patient groups and types of surgery. A summary of characteristics table will be presented for all selected anchoring reviews. Information to be presented will cover patient population characteristics, intervention including timing, comparator, type of surgery, number of studies, number of patients, ROBIS risk of bias judgement, and overall assessment of certainty of evidence using GRADE. For clinical outcomes and adverse events, the overall pooled effect size and confidence intervals and results of subgroup and sensitivity analyses (where available) will be presented. A list of outcomes reported other than those pre-specified in this protocol will be made; any extra outcomes that were not prespecified but deemed to be meaningful by the advisory panel will undergo data extraction. Narrative descriptions of any relevant emerging ongoing RCTs will be included.

The evidence maps and summary tables will provide an overview of the evidence and allow identification of evidence gaps to highlight priority for future research.

Forest plots of effect sizes for comparisons of effectiveness of oxygen therapy stratified by different subgroups will be produced as part of the panoramic meta-analyses.

## Discussion

The Royal College of Anaesthetists estimates that in a given year 1 in 20 people undergo anaesthesia for surgery in the UK [58]. As part of this procedure, patients will usually be given additional oxygen both intraoperatively and for a period post-operatively. Despite its daily and frequent use, there is significant clinical uncertainty about different aspects of perioperative oxygen therapy. There are mixed views, for example, over the potential benefits and harms of using a higher concentration of oxygen to reduce surgical site infections in certain populations, and around the use of high-flow nasal cannula oxygen therapy. There are a significant number of systematic reviews and meta-analyses looking at various aspects of perioperative oxygen therapy, but clinical uncertainty remains. A comprehensive ‘panoramic’ overview of these reviews examining the effectiveness of different approaches and what works for whom is needed to aid clinical decision making and the direction of future research.

The planned overview is ambitious given the complexity of the subject area and the large and diverse evidence base. We anticipate methodological challenges arising from different review approaches and quality and varied and potentially overlapping coverage of RCTs between existing reviews [59–61]. We have proposed a structured way to overcome these challenges, but what could be achieved will still be limited by the quality and quantity of available review and trial evidence, as well as the constraint of time and resources. We have further advocated in the proposed overview a panoramic meta-analysis approach to quantitatively examine evidence across a broad range of perioperative oxygen interventions and surgical conditions, with a view to test underlying scientific hypotheses [62]. While this approach is contrary to the convention of seeking to include homogeneous evidence within individual meta-analyses by adopting a highly focused review question/scope, we believe that the higher-level quantitative synthesis may allow further insight to be gained by comparing and contrasting different pieces of evidence in a cross-cutting subject area like this. We hope that lessons learned from undertaking this overview will help advancing the science of evidence synthesis in this respect.

## Supplementary Information

**Additional file 1.**

**Additional file 2.**

## Data Availability

All data generated or analysed during this study will be available in a published article [and its supplementary information files] once the review is completed.

## Abbreviations

ARDS: Acute respiratory distress syndrome
CDC: Centers for Disease Control and Prevention
CENTRAL: Cochrane Central Register of Controlled Trials
CPAP: Continuous positive airway pressure
DARE: The Database of Abstracts of Reviews of Effects
EMBASE: Excerpta Medica Database
EPPI: Evidence for Policy and Practice Information
FiO_2_: Fraction of inspired oxygen
GRADE: Grading of Recommendations Assessment, Development and Evaluation
HFNO: High-flow nasal oxygen
ICU: Intensive care unit
INAHTA: International Network of Agencies for Health Technology Assessment
MEDLINE: Medical Literature Analysis and Retrieval System Online
NICE: National Institute for Health and Care Excellence
NIPPV: Non-invasive positive pressure ventilation
PPC: Postoperative pulmonary complications
PRIOR: Preferred Reporting Items for Overviews of Reviews
PRISMA-P: Preferred reporting items for systematic review and meta-analysis protocol
PROSPERO: International Prospective Register of Systematic Reviews
RCTs: Randomised controlled trials
ROBIS: Risk Of Bias In Systematic Reviews
WHO: World Health Organization
