# Supplementary material for: Perioperative oxygen therapy: a protocol for an overview of systematic reviews and meta-analyses
Source: Syst Rev. 2022 Jul 12;11:140. doi: 10.1186/s13643-022-02005-3 (PMC9277880; doi:10.1186/s13643-022-02005-3)
Supplement: Supplementary file 2 — Additional file 2. [file 13643_2022_2005_MOESM2_ESM.pdf]

Perioperative Oxygen Therapy: An overview of systematic reviews and meta-analyses: Search record

Bibliographic databases: search strategies

Epistemonikos

https://www.epistemonikos.org/

Advanced Search

(title:(surg* OR operat* OR preoperativ* OR intraoperativ* OR perioperativ* OR postoperative* OR pre-operativ* OR intra-operativ* OR peri-operativ* OR post-operative* OR presurg* OR intrasurg* OR perisurg* OR postsurg* OR pre-surg* OR intra-surg* OR peri-surg* OR post-surg*) OR abstract:(surg* OR operat* OR preoperativ* OR intraoperativ* OR perioperativ* OR postoperative* OR pre-operativ* OR intra-operativ* OR peri-operativ* OR post-operative* OR presurg* OR intrasurg* OR perisurg* OR postsurg* OR pre-surg* OR intra-surg* OR peri-surg* OR post-surg*)) AND (title:(oxygen* OR hyperoxia OR "non-invasive ventilation" OR "non invasive ventilation" OR "noninvasive ventilation" OR "nasal cannula" OR "nasal cannulae" OR "nasal cannulas" OR "high flow" OR highflow OR high-flow OR HFNC OR HFOC OR "continuous positive airway pressure" OR CPAP OR "continuous positive pressure ventilation" OR CPPV OR "bi level positive airway pressure" OR "bilevel positive airway pressure" OR "bi-level positive airway pressure" OR BiPaP) OR abstract:(oxygen* OR "non-invasive ventilation" OR "non invasive ventilation" OR "noninvasive ventilation" OR "nasal cannula" OR "nasal cannulae" OR "nasal cannulas" OR "high flow" OR highflow OR high-flow OR HFNC OR HFOC OR "continuous positive airway pressure" OR CPAP OR "continuous positive pressure ventilation" OR CPPV OR "bi level positive airway pressure" OR "bilevel positive airway pressure" OR "bi-level positive airway pressure" OR BiPaP))

MEDLINE (Ovid)

Exact database segment searched: Ovid MEDLINE(R) ALL <1946 to September 13, 2021>

Search Strategy:

----

1 exp Specialties, Surgical/ (207628)

2 exp Surgical Procedures, Operative/ (3311503)

3 (surger* or surgical or surgeon* or operat* or preoperativ* or intraoperativ* or perioperativ* or postoperative* or pre-operativ* or intra-operativ* or peri-operativ* or post-operative* or presurg* or intrasurg* or perisurg* or postsurg* or pre-surg* or intra-surg* or peri-surg* or post-surg*).ti,ab,kf. (3094965)

4 1 or 2 or 3 (5123023)

5 Oxygen/ (169396)

6 exp Oxygen Inhalation Therapy/ (27158)

7 Hyperoxia/ (3989)

8 Noninvasive ventilation/ (2810)

9 Positive-Pressure Respiration/ (17710)

10 Continuous Positive Airway Pressure/ (8010)

11 (oxygen* or non invasive ventilation or noninvasive ventilation or nasal cannula* or high flow or highflow or HFNC or HFOC or continuous positive airway pressure or CPAP or continuous positive pressure ventilation or CPPV or bi level positive airway pressure or bilevel positive airway pressure or BiPaP).ti,ab,kf. (625166)

12 5 or 6 or 7 or 8 or 9 or 10 or 11 (710401)

13 4 and 12 (102058)

14 (metaanalys* or meta analys* or meta-analys* or NMA* or MAIC* or indirect comparison* or indirect treatment comparison* or mixed treatment comparison*).mp. (247161)

15 ((systematic* adj3 (review* or overview* or search or literature)) or umbrella review*).mp. (271732)

16 (technology assessment* or HTA or HTAs or technology overview* or technology appraisal*).mp. (17024)

17 14 or 15 or 16 (406117)

18 13 and 17 (1396)

19 limit 13 to (meta analysis or "systematic review") (951)

20 18 or 19 (1396)

Embase (Ovid)

Database: Embase Classic+Embase <1947 to 2021 Week 36>

Search Strategy:

----

1 exp surgery/ (5639002)

2 exp surgeon/ (177960)

3 perioperative medicine/ (289)

4 (surger* or surgical or surgeon* or operat* or preoperativ* or intraoperativ* or perioperativ* or postoperative* or pre-operativ* or intra-operativ* or peri-operativ* or post-operative* or presurg* or intrasurg* or perisurg* or postsurg* or pre-surg* or intra-surg* or peri-surg* or post-surg*).ti,ab,kw. (4262932)

5 1 or 2 or 3 or 4 (7197759)

6 oxygen/ (256042)

7 oxygen therapy/ (37316)

8 hyperoxia/ (10603)

9 exp noninvasive ventilation/ (15524)

10 exp positive pressure ventilation/ (10805)

11 (oxygen* or non invasive ventilation or noninvasive ventilation or nasal cannula* or high flow or highflow or HFNC or HFOC or continuous positive airway pressure or CPAP or continuous positive pressure ventilation or CPPV or bi level positive airway pressure or bilevel positive airway pressure or BiPaP).ti,ab,kw. (797248)

12 6 or 7 or 8 or 9 or 10 or 11 (907180)

13 5 and 12 (165461)

14 (metaanalys* or meta analys* or meta-analys* or NMA* or MAIC* or indirect comparison* or indirect treatment comparison* or mixed treatment comparison*).mp. (357848)

15 ((systematic* adj3 (review* or overview* or search or literature)) or umbrella review*).mp. (432167)

16 (technology assessment* or HTA or HTAs or technology overview* or technology appraisal*).mp. (26895)

17 14 or 15 or 16 (617074)

18 13 and 17 (2863)

Cochrane Database of Systematic Reviews (Wiley)

Search date: 15/09/2021

ID Search Hits

#1 MeSH descriptor: [Specialties, Surgical] explode all trees 1942

#2 MeSH descriptor: [Surgical Procedures, Operative] explode all trees 123492

#3 (surg* or operat* or preoperativ* or intraoperativ* or perioperativ* or postoperative* or pre-operativ* or intra-operativ* or peri-operativ* or post-operative* or presurg* or intrasurg* or perisurg* or postsurg* or pre-surg* or intra-surg* or peri-surg* or post-surg*):ti,ab,kw 321509

#4 #1 or #2 or #3 356935

#5 MeSH descriptor: [Oxygen] this term only 5257

#6 MeSH descriptor: [Oxygen Inhalation Therapy] explode all trees 1650

#7 MeSH descriptor: [Hyperoxia] this term only 211

#8 MeSH descriptor: [Noninvasive Ventilation] this term only 295

#9 MeSH descriptor: [Positive-Pressure Respiration] this term only 1564

#10 MeSH descriptor: [Continuous Positive Airway Pressure] this term only 1173

#11 (oxygen* or "non invasive ventilation" or "noninvasive ventilation" or (nasal next cannula*) or "high flow" or highflow or HFNC or HFOC or "continuous positive airway pressure" or CPAP or "continuous positive pressure ventilation" or CPPV or "bi level positive airway pressure" or "bilevel positive airway pressure" or BiPaP):ti,ab,kw 59119

#12 #5 or #6 or #7 or #8 or #9 or #10 or #11 59619

#13 #4 and #12 17322

Cochrane Reviews: 220

Cochrane Protocols: 3

PROSPERO

https://www.crd.york.ac.uk/prospero/

Search date: 15/09/2021

#1 (surg* OR operat* OR preoperativ* OR intraoperativ* OR perioperativ* OR postoperative* OR pre-operativ* OR intra-operativ* OR peri-operativ* OR post-operative* OR presurg* OR intrasurg* OR perisurg* OR postsurg* OR pre-surg* OR intra-surg* OR peri-surg* OR post-surg*):TI,CS,HA,KW,PA,RQ 24143

#2 (oxygen* OR non-invasive ventilation OR non invasive ventilation OR noninvasive ventilation OR nasal cannula OR nasal cannulas OR high flow OR highflow OR high-flow OR HFNC OR HFOC OR continuous positive airway pressure OR CPAP OR continuous positive pressure ventilation OR CPPV OR bi level positive airway pressure OR bilevel positive airway pressure OR bi-level positive airway pressure OR BiPaP):TI,KW,RQ 1031

#3 (oxygen* OR non-invasive ventilation OR non invasive ventilation OR noninvasive ventilation OR nasal cannula OR nasal cannulas OR high flow OR highflow OR high-flow OR HFNC OR HFOC OR continuous positive airway pressure OR CPAP OR continuous positive pressure ventilation OR CPPV OR bi level positive airway pressure OR bilevel positive airway pressure OR bi-level positive airway pressure OR BiPaP):IV 1011

#4 #2 OR #3 1484

#5 #1 AND #4 325

HTA Database (INAHTA)

https://database.inahta.org/

Search date: 15/09/2021

(((oxygen* or "non invasive ventilation" or "non-invasive ventilation" or "noninvasive ventilation" or "nasal cannula" or "nasal cannulas" or "nasal cannulae" or "high flow" or "high-flow"or highflow or HFNC or HFOC or "continuous positive airway pressure" or CPAP or "continuous positive pressure ventilation" or CPPV or "bi level positive airway pressure" or "bi-level positive airway pressure" or "bilevel positive airway pressure" or BiPaP)[abs]) OR ((oxygen* or "non invasive ventilation" or "non-invasive ventilation" or "noninvasive ventilation" or "nasal cannula" or "nasal cannulas" or "nasal cannulae" or "high flow" or "high-flow"or highflow or HFNC or HFOC or "continuous positive airway pressure" or CPAP or "continuous positive pressure ventilation" or CPPV or "bi level positive airway pressure" or "bi-level positive airway pressure" or "bilevel positive airway pressure" or BiPaP)[title]) OR ("Continuous Positive Airway Pressure"[mh]) OR ("Positive-Pressure Respiration"[mh]) OR ("Noninvasive Ventilation"[mh]) OR ("Hyperoxia"[mh]) OR ("Oxygen Inhalation Therapy"[mhe]) OR ("Oxygen"[mh])) AND ((((surger* or surgical or surgeon* or operat* or preoperativ* or intraoperativ* or perioperativ* or postoperativ* or pre-operativ* or intra-operativ* or peri-operativ* or post-operative* or "pre operative" or "intra operative" or "peri operative" or "post operative" or presurg* or intrasurg* or perisurg* or postsurg* or pre-surg* or intra-surg* or peri-surg* or post-surg*)[abs]) OR ((surger* or surgical or surgeon* or operat* or preoperativ* or intraoperativ* or perioperativ* or postoperativ* or pre-operativ* or intra-operativ* or peri-operativ* or post-operative* or "pre operative" or "intra operative" or "peri operative" or "post operative" or presurg* or intrasurg* or perisurg* or postsurg* or pre-surg* or intra-surg* or peri-surg* or post-surg*)[title])) OR ("Surgical Procedures, Operative"[mhe]) OR ("Specialties, Surgical"[mhe]))

93

DARE archives (CRD)

Search date: 15/09/2021

1	MeSH DESCRIPTOR Specialties, Surgical EXPLODE ALL TREES	375
2	MeSH DESCRIPTOR Surgical Procedures, Operative EXPLODE ALL TREES	16740
3	(surg* OR operat* OR preoperativ* OR intraoperativ* OR perioperativ* OR postoperative* OR pre-operativ* OR intra-operativ* OR peri-operativ* OR post-operative* OR presurg* OR intrasurg* OR perisurg* OR postsurg* OR pre-surg* OR intra-surg* OR peri-surg* OR post-surg*)	19957
4	#1 OR #2 OR #3	25084
5	MeSH DESCRIPTOR Oxygen	105
6	MeSH DESCRIPTOR Oxygen Inhalation Therapy EXPLODE ALL TREES	172
7	MeSH DESCRIPTOR Hyperoxia	3
8	MeSH DESCRIPTOR Noninvasive Ventilation	17

9	MeSH DESCRIPTOR Positive-Pressure Respiration	97
10	MeSH DESCRIPTOR Continuous Positive Airway Pressure	122
11	(oxygen* or non invasive ventilation or noninvasive ventilation or nasal cannula* or high flow or highflow or HFNC or HFOC or continuous positive airway pressure or CPAP or continuous positive pressure ventilation or CPPV or bi level positive airway pressure or bilevel positive airway pressure or BiPaP)	1340
12	#5 OR #6 OR #7 OR #8 OR #9 OR #10 OR #11	1384
13	#4 AND #12	392
14	(#4 AND #12) IN DARE	218
